# Exploring the predictive factors for depression among hemodialysis patients: a case-control study

**DOI:** 10.1192/bjo.2021.165

**Published:** 2021-06-18

**Authors:** Syed Muhammad Jawad Zaidi, Mehwish Kaneez, Hamza Waqar Bhatti, Shanzeh Khan, Shafaq Fatima, Muhammad Hamza, Mishal Fatima

**Affiliations:** 1 Final Year MBBS Student, Rawalpindi Medical University; 2 Rawalpindi Medical University

## Abstract

**Aims:**

Depression remains an exceedingly ubiquitous entity that significantly depreciates the quality of life and disease prognosis among end-stage renal disease (ESRD) patients. Even though the deleterious effects of depression on ESRD patients are well-established in the literature, the predictive factors that predispose such patients to depression need to be explored. Our study thus aims to gauge these factors and create a predictive model for optimal psychiatric and medical management of such patients.

**Method:**

All ESRD patients with a disease duration of at least one year underwent a complete psychiatric evaluation based on DSM-V guidelines preceded by a cognitive evaluation by Mini-Mental State Examination (MMSE). A total of 73 patients diagnosed with moderate to severe major depressive disorder were selected as cases. Patients suffering from recurrent psychotic episodes, having a past or family history of psychiatric illness, being already treated for depression, having any substance abuse (current or past), were excluded from the study. Following the similar guidelines, and exclusion criteria, 146 patients (two controls for each case) having no depression were selected as controls. The cases and controls were studied and matched for a myriad of sociodemographic factors. The various risk factors for depression were evaluated using univariate and multivariate binary logistics analysis.

**Result:**

The significant risk factors for depression among hemodialysis patients were age (OR = 1.79, CI = 0.47–3.81), comorbidities (OR = 2.13, CI = 0.51–3.96), duration of renal disease (OR = 2.54, CI 0.63–4.28), duration of hemodialysis (OR = 2.36, CI = 0.89–4.11), unemployment (OR = 2.33, CI = 0.79–3.88), and being unmarried (OR = 1.93, CI = 0.44–3.53). Prospect of survival, financial instability, social stigmatization, and effect of comorbidities on ESRD were major concerns for the cases that attributed to their depressive symptoms.

**Conclusion:**

The factors that herald the onset of depression among hemodialysis patients include increasing age, presence of comorbidities, unemployment being unmarried, and increasing duration of hemodialysis. These factors will aid the clinicians to identify high-risk patients that require psychiatric consultation. We recommend prompt psychiatric intervention (pharmacologic or non-pharmacologic) and appropriate patient counseling so that the depressive symptoms can be alleviated and dismal disease prognosis can be prevented among such high-risk patients.

